# Comparison of three‐dimensional analysis and stereological techniques for quantifying lithium‐ion battery electrode microstructures

**DOI:** 10.1111/jmi.12389

**Published:** 2016-03-21

**Authors:** OLUWADAMILOLA O. TAIWO, DONAL P. FINEGAN, DAVID S. EASTWOOD, JULIE L. FIFE, LEON D. BROWN, JAWWAD A. DARR, PETER D. LEE, DANIEL J.L. BRETT, PAUL R. SHEARING

**Affiliations:** ^1^ The Electrochemical Innovation Lab, Department of Chemical EngineeringUniversity College LondonLondonWC1E 7JEU.K.; ^2^ Manchester X‐Ray Imaging FacilitySchool of Materials, University of ManchesterManchester M13 9PLU.K.; ^3^ Swiss Light SourcePaul Scherrer Institut5232 Villigen, PSISwitzerland; ^4^ Department of ChemistryUniversity College London20 Gordon StreetLondonWC1H 0AJU.K.

**Keywords:** Image quantification, lithium‐ion battery, microstructural characterization, stereology, X‐ray tomography, 3‐D image analysis

## Abstract

Lithium‐ion battery performance is intrinsically linked to electrode microstructure. Quantitative measurement of key structural parameters of lithium‐ion battery electrode microstructures will enable optimization as well as motivate systematic numerical studies for the improvement of battery performance. With the rapid development of 3‐D imaging techniques, quantitative assessment of 3‐D microstructures from 2‐D image sections by stereological methods appears outmoded; however, in spite of the proliferation of tomographic imaging techniques, it remains significantly easier to obtain two‐dimensional (2‐D) data sets. In this study, stereological prediction and three‐dimensional (3‐D) analysis techniques for quantitative assessment of key geometric parameters for characterizing battery electrode microstructures are examined and compared. Lithium‐ion battery electrodes were imaged using synchrotron‐based X‐ray tomographic microscopy. For each electrode sample investigated, stereological analysis was performed on reconstructed 2‐D image sections generated from tomographic imaging, whereas direct 3‐D analysis was performed on reconstructed image volumes. The analysis showed that geometric parameter estimation using 2‐D image sections is bound to be associated with ambiguity and that volume‐based 3‐D characterization of nonconvex, irregular and interconnected particles can be used to more accurately quantify spatially‐dependent parameters, such as tortuosity and pore‐phase connectivity.

## Lay description

There is a strong link between electrode microstructure and battery performance within lithium‐ion batteries. Quantitatively assessing these inherently 3‐D electrode microstructures will help provide data for numerical studies to help improve battery performance. With the rapid development of 3‐D imaging techniques, quantitative assessment of 3‐D microstructures from 2‐D image sections by stereological methods appears outmoded; however, in spite of the proliferation of tomographic imaging techniques, it remains significantly easier to obtain 2‐D data sets. In this study, stereological prediction and 3‐D analysis techniques for quantitative assessment of key geometric parameters for characterizing battery electrode microstructures are examined and compared. The analysis showed that geometric parameter estimation using 2‐D image sections is bound to be associated with ambiguity and that volume‐based 3‐D characterization of nonconvex, irregular and interconnected particles can be used to more accurately quantify spatially‐dependent parameters, such as tortuosity and pore‐phase connectivity.

## Introduction

Reactions that take place within lithium‐ion batteries are supported by porous composite electrodes possessing complex microstructures, and as with all functional materials, there is a direct relationship between electrode microstructure and battery performance. A quantitative understanding of microstructure and transport pathways within lithium ion battery electrodes is crucial for improving their design, manufacture, performance and durability. To this end, characterization techniques for revealing all relevant, detailed microstructural morphology and for assessing quantitative geometric parameters are essential.

Three‐dimensional (3‐D) microstructures can be characterised using two principal approaches: the first is by stereological methods (DeHoff & Rhines, 1961; DeHoff & Rhines, 1968; Underwood, 1970; Mouton, 2002), which take measurements from planar two‐dimensional (2‐D) image slices through a sample material (typically obtained using 2‐D cross‐sectional microscopy), and extrapolating the results to the geometric parameters of the 3‐D structure using statistical approaches and image analysis. The second approach is by direct viewing and measurement of 3‐D datasets obtained by tomographic imaging of the material of interest or serial sectioning techniques. X‐ray tomography (Chen‐Wiegart *et al*., 2012; Shearing *et al*., 2012; Ebner *et al*., 2013) and FIB‐SEM tomography (Ender *et al*., 2011; Wilson *et al*., 2011; Hutzenlaub *et al*., 2012) are the most commonly applied imaging techniques for obtaining complete microstructural models of lithium‐ion battery electrodes (Shearing *et al*., 2012).

Although stereological procedures require relatively less experimental and computational effort, microstructural investigations using these methods are often inconclusive, particularly when attempting to characterize structural quantities that rely on phase connectivity in 3‐D. In contrast, 3‐D microstructural analysis from tomographic imaging can be time consuming and may require access to advanced tomography equipment.

This paper will examine and compare both stereological prediction and 3‐D analysis techniques for quantitative measurements of key geometric parameters that characterise lithium‐ion battery electrode microstructures. In this work, lithium‐ion battery electrode samples were imaged using synchrotron‐based X‐ray tomography, and for each electrode sample investigated, stereological analysis was performed on reconstructed 2‐D image slices extracted from tomographic imaging whereas direct 3‐D analysis was performed on reconstructed image volumes.

## Materials and methods

### X‐ray tomography and image analysis

Three commercial lithium‐ion battery electrodes materials: lithium cobalt oxide (LCO), lithium manganese oxide (LMO) and graphite (MTI Corporation, California, USA) were imaged using synchrotron radiation X‐ray tomographic microscopy at the TOMCAT beamline of the Swiss Light Source (Paul Scherrer Institut, Villigen, Switzerland) (Stampanoni *et al*., 2006). Small sections of the printed electrode materials were cut to a size which fit into the user‐defined field of view, set to 1.6 × 1.4 mm^2^ for this experiment.

A parallel monochromatic beam was used in absorption‐contrast imaging mode, with the beam energy set to 10.5 keV for the graphite electrode and 18 keV for both LCO and LMO materials. For each tomographic scan, 1501 projections were acquired during a 180° rotation of the sample about its long axis, through angular steps of 0.12° with an exposure time of 200 ms for each projection image. The X‐rays illuminated a 20‐μm‐thick LuAG:Ce scintillator, producing visible light which was focused onto a PCO.Edge camera, providing an effective pixel size of 0.65 μm. Tomographic reconstruction of the acquired projection images was performed using the gridrec algorithm (Marone & Stampanoni, 2012) after application of flat‐ and dark‐field corrections to account for nonuniformity in the incident beam and detector response, respectively. Table 1 is a summary of the parameters used to image the different samples.

**Table 1:** Tomography acquisition parameters for each sample

Scan parameters	LCO	LMO	Graphite
Beam energy (keV)	18	18	10.5
Number of projections	1501		
Radiograph exposure time (ms)	200		
Rotation range (°)	[0, 180]		
Effective voxel size (nm^3^)	650 × 650 × 650		

**Figure 1:**
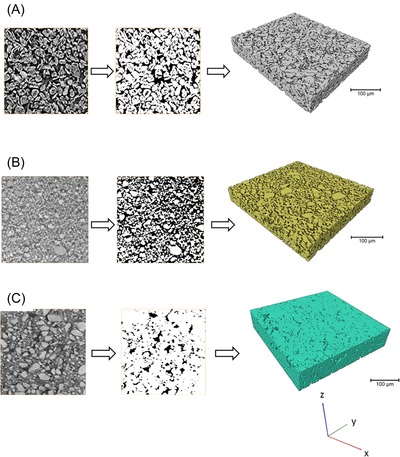
Transformation of greyscale 2‐D image to binary image 2‐D and ultimately a volume rendering of the (A) graphite, (B) LCO and (C) LMO electrode samples respectively, where white represents the solid phase and black represents the pore/electrolyte phase.

Image preprocessing and volume rendering of the resulting reconstructed volumes (Fig. 1) was carried out using the Avizo Fire software package (Avizo Fire 8.0, FEI Visualization Sciences Group, Mérignac Cedex, France). For each acquired tomographic volume (consisting of a stacked sequence of 2‐D grayscale images), a region‐of‐interest (ROI) was extracted for subsequent analysis. After ROI extraction, a nonlocal means smoothing filter was applied to the greyscale image sequences for image smoothing and denoising, followed by threshold segmentation which utilizes the greyscale histogram of the tomographic images to separate out the solid phase from the pore phase, creating a binary image.

### Quantification of microstructural parameters

In order to compare stereological and direct 3‐D quantification procedures, some common geometric parameters were analysed, namely: pore volume fraction, volume‐specific surface area, geometric tortuosity and pore radius. Stereological or 2‐D‐based predictions of 3‐D microstructural parameters are usually obtained by performing quantitative image analysis on 2‐D image sections—this could be done using a single 2‐D cross‐sectional image slice or a statistical sampling of a few image slices. In this work, however, we perform stereological analysis on every 2‐D cross‐sectional slice from each of the tomographic image sequences; thus, for each calculated parameter, a slicewise distribution across the sample volume is generated over which an average is taken. With direct 3‐D quantification, microstructural parameters are extracted from the tomographic image volume. In this approach, image sections (actual or computer‐generated, depending on the imaging technique employed) can be read into a computer as a 3‐D volume using digital image analysis (as a matrix of volume pixels or voxels).

- *Pore volume fraction* (ϕ): This is the volume fraction of the pore phase with respect to the total volume of sample porous material being analysed. Pore volume fraction can be predicted stereologically using Delesse's principle (DeHoff & Rhines, 1968; Underwood, 1970) which estimates the volume fraction of a component/phase of interest within a 3‐D object from the area fraction of that component within a 2‐D cross‐section through that object ($A_{A}$), that is, ϕ = $A_{A}$. Area fraction calculations were performed on each 2‐D cross‐sectional slice generated from the tomography image sequences using a pixel counting approach as the ratio of the total number of pixels in the pore phase to the total number of pixels in the analysed image slice. Pore volume fraction was calculated directly from the tomographic image volumes, using a voxel counting approach, as the fraction of cubic voxels that make up the pore phase within the analysed 3‐D volume of interest.
- *Volume‐specific surface area (S_V_)*: For a given interface or phase boundary within an imaged sample volume, $S_{V}$ can be defined as the ratio of the total surface area of that interface type in the sample to the sample volume. $S_{V}\;$for the solid phase/porosity interface in the lithium‐ion battery electrode samples was predicted using a stereological approach which requires the measurement of $L_{A},$ the perimeter length of the total phase boundary observed within the given 2‐D cross‐section per unit area of the image cross‐section (DeHoff & Rhines, 1968; Underwood, 1970; Mouton, 2002), that is,
  (1) $$S_{V}=\;\frac{4}{\pi }\left(L_{A}\right).$$
The  variable SVwas directly quantified in 3‐D as the ratio of the total 3‐D surface area of the sample solid phase/porosity interface to the total analysed sample volume. The surface area of the solid phase/porosity interface was calculated as the area of a triangular surface mesh approximation of the solid phase/porosity interface. This was performed using the ‘Surface Area‐Volume’ quantification module in Avizo Fire software. Here, a marching cubes algorithm (Lorensen & Cline, 1987) is applied to the segmented image to generate a triangulated surface. In order to capture phase interfaces, the marching cube algorithm in Avizo Fire allows for generating interfaces between multiple segmented phases, and can use subvoxel weights for surface smoothing. A constrained surface smoothing was applied using subvoxel weights and with a smoothing extent of 5 in the surface generation step. For this surface area calculation approach, mesh refinement was done and validated against the analytical solution of model image samples.

iii. *Geometric tortuosity (τ)*: Tortuosity (τ) is a term commonly used to describe the complexity and interconnectedness of transport paths in porous media. In lithium‐ion batteries, the tortuosity of the pore phase describes the influence of electrode morphology on lithium‐ion transport with the electrolyte. In the literature, the term has been defined from diffusion transport point of view (as tortuosity factor, *τ^2^*) and from a geometrical point of view (Epstein, 1989). Geometrically, τ can be defined in 2‐D using the *arc‐chord ratio* which is the ratio of the effective length of a curve (*L_e_*) to the length of the straight line between the curve's end points (*L*), that is,
  (2) $$\tau \;=\;\frac{L_{e}}{L}.$$
  However, extending this 2‐D definition to branching 3‐D networks, τ can be extracted between a source plane and a destination plane within a porous volume, and defined for each location x on the destination plane (i.e. in the *x*, *y* or *z* directions) where it intersects with the 3‐D network:
  (3) $$\tau \;\left(x\right)=\;\frac{L_{e}\left(x\right)}{L\left(x\right)}.$$
  where $L_{e}\left(x\right)$ represents the shortest path length through the selected phase from the source plane to the location x and *L*(x) represents the shortest path through any phase in the sample volume from the source to destination planes. With each of the examined 3‐D volumes, τ for the pore phase was calculated along the *x, y* and *z* axes.
  Here, the tortuosity distributions were calculated based on source backtracking, which uses the fast marching algorithm on the binarized tomographic image sequence to draw a source‐distance map of the propagating front. This source map was then used to reconnect any connected component to the nearest geodesic path to the starting boundaries. The reader can find more information about using the FMM method to create distance distribution maps in *Jørgensen et al*. (2011).

iv. *Pore radius (*σ*)*: Within 3‐D porous media, the pore size distribution is usually characterized by a mean pore size. Stereologically, such media can be characterized by a mean pore size that is not derived from a pore size distribution but from using the concept of mean intercept length (Underwood, 1970). From this, the stereologically predicted mean pore radius ($\sigma_{2D}$) can be defined by the following relation:
  (4) $$\sigma_{2D}=\frac{2V_{V}}{S_{V}},$$
  where *V_V_* and *S_V_* are the volume fraction and volume‐specific surface area of the pore phase, respectively.
  From a geometrical point of view, the pore radius for any section of the pore path in the network can be defined as the radius of the largest sphere that can be locally inscribed into that section of the network (Jørgensen *et al*., 2011). For the 3‐D pore size quantification, the mean pore radius is calculated by taking the average of a generated pore radius distribution and various methods exist for extracting pore radius distributions from 3‐D image volumes; however, in this work, we compare these four methods:

● *Medial axis based method using fast marching (MA‐FM)*: This method first extracts the medial axis (or skeleton) of the pore network by performing a skeletonization on the 3‐D pore image with the aid of a multistencil fast‐marching algorithm (Hassouna & Farag, 2007), and then takes the pore radius from every point along the resulting topological skeleton as the distance to the nearest pore phase boundary.
● *Medial axis‐based method using distance‐ordered homotopic thinning (DOHT)*: This method (Pudney, 1998) also calculates pore radius as the distance to the nearest pore phase boundary to the pore skeleton, but performs the skeletonization by combining morphological thinning and distance map based techniques. First, a chamfer distance map of the 3‐D pore space is computed, which contains the shortest distance of each point in the pore space to the pore phase boundary, and then the resulting distance map is used to guide the thinning algorithm. This approach was implemented using the Avizo Fire 8.0 ‘Auto‐Skeleton’ module.
● *Successive morphological opening method (SMO)*: This method applies successive morphological opening on the 3‐D pore space with spherical structuring elements (SEs) of increasing size (Daïan *et al*., 2004; Dupuy *et al*., 2011; Yang *et al*., 2014). Here, opening refers to the process of first dilating, and then eroding an image using a SE. Hence, if a spherical SE of 1 pixel radius is used to ‘open’ the 3‐D pore image, the resulting image will only have pores larger than 1 pixel in radius. In this method, the opening operation is repeated with increasing SE size until there are no more pores existing in the 3‐D pore volume. For each opening operation, the accumulated volume fraction of pores larger than a certain SE of radius *r* can be calculated as
  (5) $$\upsilon \left(r\right)\;=\;1-\frac{\beta \left(r\right)}{\beta },$$
  where β is the sum of the pore phase voxels in the initial 3‐D volume, β(r) is the sum of pore phase voxels in the 3‐D volume after the morphological opening operation with a SE of radius *r*. From this, the volume fraction of pores with a certain radius can be obtained by the difference of two successively accumulated volume fractions. The pore radius obtained using this method is called equivalent radius and it is equivalent to the radius of the maximal inscribed sphere in the pore.
● *Continuous pore size distribution method (CPSD)*: The ‘continuous pore size distribution’ method (Münch & Holzer, 2008; Holzer *et al*., 2011) is based on an algorithm which measures the pore volume that can be covered with a sphere of given radius. This algorithm can also be seen to represent, in this sense, the simulation of a pressure induced intrusion with a poorly wetting fluid (e.g. mercury intrusion). Here, a continuous pore size distribution is obtained by incrementally reducing the radius and thereby filling a larger volume: by decreasing the radius, more constricted areas such as pore bottle‐necks and narrow corners can be intruded. A cumulative PSD is obtained as a result by relating the incrementally filled volume with corresponding radii, which can then be normalized.

### Representative volume element analysis

**Figure 2:**
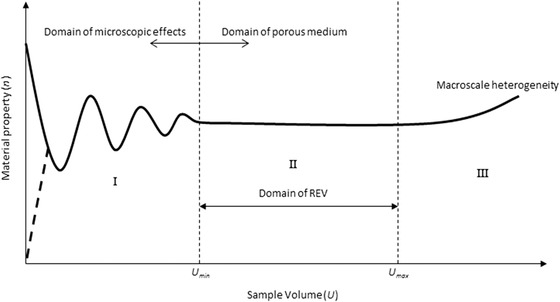
Conceptual schematic representing the idealized relationship between material property (*n*) and the sample volume (*U*) and showing the representative volume element region. Adapted from Costanza‐Robinson *et al*. (2011).

To allow ease of manipulation for the direct 3‐D parameter quantification, the largest possible cuboid volume was cropped from each tomographic dataset (Table 2); however, to ensure that these cropped electrode volumes were large enough to be representative of the macroscopic properties of the respective bulk electrode volumes, representative volume element (RVE) analysis was performed. The RVE is usually regarded as a volume of heterogeneous material which effectively includes a sampling of all microstructural heterogeneities present and is sufficiently large to be statistically representative for the entire structure; further, the size of the RVE also depends on the investigated morphological or physical properties (Kanit *et al*., 2003). In practice, the RVE can be estimated deterministically by performing a systematic analysis of the influence of volume size on the overall geometric or physical properties of interest (Bernard *et al*., 2005; Costanza‐Robinson *et al*., 2011), with the minimum RVE given by the size of the volume for which the fluctuations in the effective property become insignificant, seen as a distinct plateau (i.e. the onset of region II in Fig. 2). However, for real heterogeneous systems, the presence of region II may be difficult to delineate with confidence due to spatial variability (Zhang *et al*., 2000; Baveye *et al*., 2002), and as a result, we adopt a methodology based on a study by Li *et al*. (2009) who point out that the RVE for a given material property can be determined for a required error, besides visual assessment of whether each RVE plot exhibited a distinct plateau or not. Here, the selected volume of interest for each analysed electrode was deemed a representative volume element if the absolute value of the relative error (*ε_r,RVE_*) in the measured material parameter was less than or equal 2 %.

**Table 2:** Volume dimensions of the analysed electrode samples

Electrode sample	Analysed volume dimensions *X* × *Y* × *Z* voxel^3^ (μm^3^)
LCO	744 × 1140 × 75 (483.6 × 741.0 × 48.8)
Graphite	1022 × 1176 × 85 (664.3 × 764.4 × 55.3)
LMO	990 × 1125 × 90 (643.5 × 731.3 × 58.5)

For the RVE analysis, the ROI extracted for each electrode sample was split into subvolumes where each subvolume was obtained by increasing the *x* and *y* lengths from an initial cuboidal volume selected from a vertex of the image, whilst keeping the *z*‐length (i.e. electrode thickness) fixed for all subvolumes. This was followed by computing the value of each microstructural parameter for the different incremental subvolumes in attempt to realise converging RVE plots.

## Results and discussion

**Figure 3:**
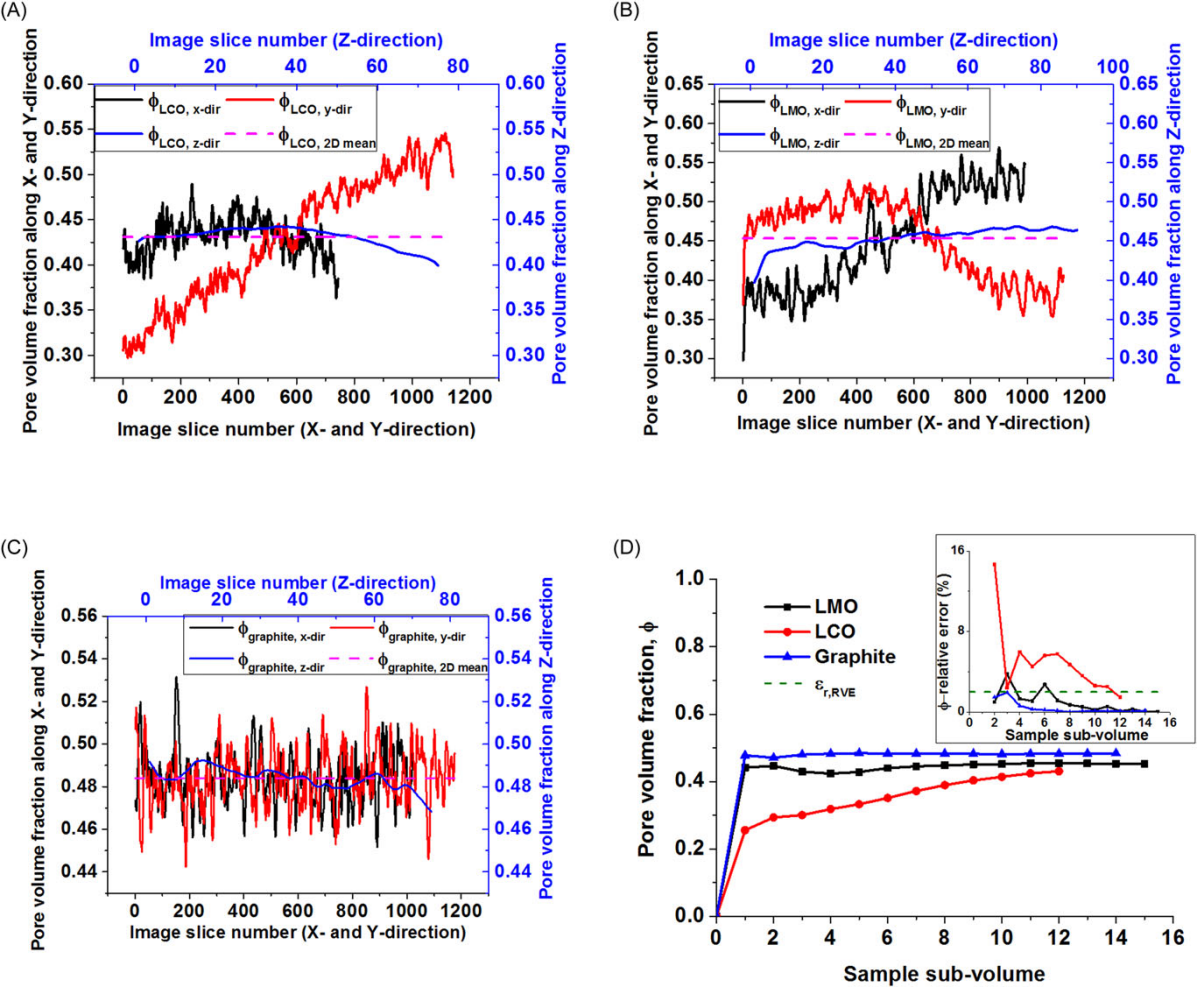
Pore volume fraction profiles for (A) LCO, (B) LMO and (C) graphite electrode samples showing variation in slicewise pore volume fraction along the *x*, *y* and *z* directions. (D) Evolution of 3‐D pore volume fraction (main) and the relative errors (inset) versus subvolume size for each electrode sample.

**Figure 4:**
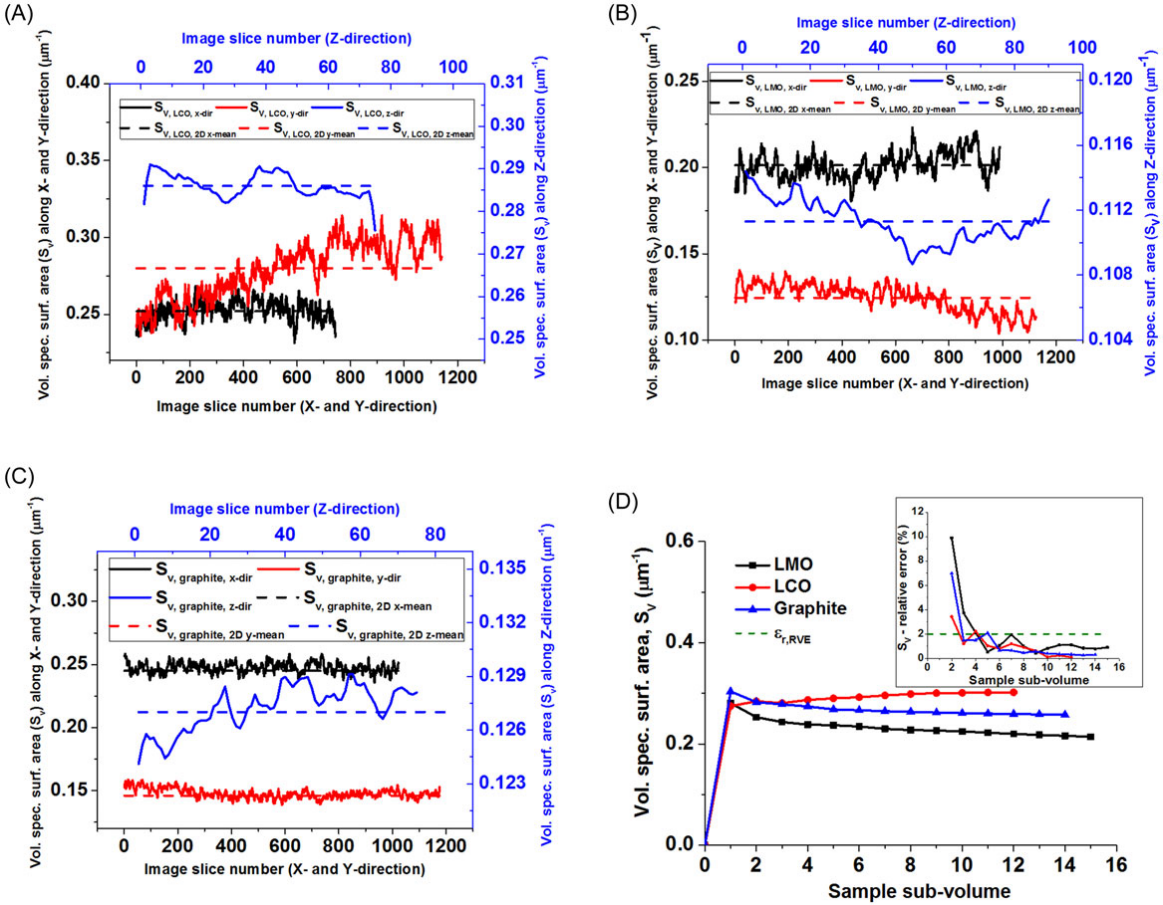
Volume‐specific surface area profiles for (A) LCO, (B) LMO and (C) graphite electrode samples showing variation in slicewise volume‐specific surface area along the *x*, *y* and *z* directions. (D) Evolution of 3‐D volume‐specific surface area (main) and the relative errors (inset) versus subvolume size for each electrode sample.

In this work, we compare the geometric quantification of battery electrode microstructural parameters using both stereological and direct 3‐D measurement approaches. Note that each reconstructed 2‐D image section is a plane of single voxel thickness. Parameter calculation using the stereological approach was performed on 2‐D image sections to yield slicewise distributions and RVE analysis was performed on the 3‐D reconstructed volumes to ensure bulk representation of the calculated parameters in three dimensions. Based on a 2 % relative error criterion, the profiles for pore volume fraction, volume‐specific surface area, as well as geometric tortuosity along the *x*, *y* and *z* directions presented in Figures 3(D), 4(D) and 6, respectively show that the three electrode sample volumes yield parameter values that are representative of the bulk electrode volume. The graphite and LMO samples reach the minimum RVE at a much smaller subvolume than the LCO sample, which can be attributed to lesser microscopic heterogeneity effects on the microstructural parameters in both graphite and LMO sample volumes.

### Pore volume fraction

Figures 3(A–C) show the slicewise pore volume fraction profile for each of the three examined battery electrodes along each orthogonal direction. For each electrode sample, the mean pore volume fraction and standard deviation (SD) in each direction were also calculated over the 2‐D image sections. As expected, averaging the entire slicewise 2‐D pore volume fraction distribution along each axis yields stereological mean pore volume fraction values (*Mean 2‐D*) which are identical to the value obtained from a 3‐D reconstruction of the pore phase, as seen in Table 3, but the pore volume fraction standard deviation varies in each direction. It can also be seen that stereological prediction of pore volume fraction using 2‐D image sections along each axis yields a pore volume fraction profile which displays significant variation, thus highlighting localised microstructural heterogeneities. When compared to the pore volume fraction measured in 3‐D, all three electrodes showed larger maximum percentage underestimation (% UE) and maximum percentage overestimation (% OE) in the *x* and *y* directions than in the *z* direction. Moreover, the results further indicate that basing pore volume fraction calculations for a 3‐D porous material with a heterogeneous microstructure on the area fraction of just a single planar section of such material could be misleading.

**Table 3:** Comparison of pore volume fraction obtained by stereological predictions and 3‐D analysis

Pore volume fraction, ϕ							
Sample	3‐D	Mean 2‐D	SD 2‐D	Min 2‐D	% UE	Max 2‐D	% OE
LCO	0.431	*x*‐dir: 0.431	0.021	0.363	15.67	0.490	13.69
		*y*‐dir: 0.431	0.070	0.297	30.96	0.546	26.69
		*z*‐dir: 0.431	0.011	0.399	7.26	0.442	2.73
Graphite	0.484	*x*‐dir: 0.484	0.013	0.452	6.67	0.531	9.81
		*y*‐dir: 0.484	0.013	0.443	8.60	0.527	8.84
		*z*‐dir: 0.484	0.005	0.468	3.24	0.492	1.73
LMO	0.453	*x*‐dir: 0.453	0.062	0.298	34.36	0.569	25.58
		*y*‐dir: 0.453	0.047	0.353	22.07	0.528	16.33
		*z*‐dir: 0.453	0.013	0.397	12.47	0.469	3.38

### Volume‐specific surface area

Figure 4 shows the slicewise volume‐specific surface area profiles generated using the stereological relation shown in Eq. (1) for each of the three examined battery electrodes along each orthogonal direction. Unlike the pore volume fraction results, it can be seen for all three samples that the mean stereological volume‐specific surface area values obtained along each axis direction differ from each other and also from the mean volume‐specific surface value obtained from 3‐D volume analysis; for instance, when compared with the mean volume‐specific surface area obtained from 3‐D measurement shown in Table 4, a difference of up to 52 % was found in the LMO electrode sample. This error can be associated with the fact that the stereological method does not account for how each image slice interacts with the next when estimating the length of the pore‐solid phase boundary length. Furthermore, in the LMO and graphite samples, the 2‐D image sections extracted in the *x* direction yield a slicewise profile with higher volume‐specific surface area values than the profiles from the 2‐D sections in the *y* and *z* directions. This could be attributed to the anisotropic or nonspherical nature of the electrode particle shapes and/or to particle‐to‐particle ordering and orientation within the electrode samples as a result of electrode calendaring or the electrode manufacturing process; this could then lead to a variation in the length of the particle–porosity phase boundary that would be captured in 2‐D sections along a given orthogonal direction.

**Table 4:** Comparison of volume‐specific area obtained by stereological predictions and 3‐D analysis

Volume‐specific surface area, $S_{V}$							
Sample	3‐D (μm^−1^)	Mean 2‐D (μm^−1^)	SD 2‐D (μm^−1^)	Min 2‐D (μm^−1^)	% UE	Max 2‐D (μm^−1^)	% O.E.
LCO	0.302	*x*‐dir: 0.252	0.007	0.231	23.38	0.268	11.17
		*y*‐dir: 0.280	0.018	0.236	21.83	0.314	4.08
		*z*‐dir: 0.286	0.003	0.276	8.77	0.291	3.65
Graphite	0.258	*x*‐dir: 0.248	0.004	0.236	8.64	0.258	0.21
		*y*‐dir: 0.148	0.004	0.139	46.26	0.158	38.49
		*z*‐dir: 0.127	0.001	0.124	51.89	0.129	49.95
LMO	0.214	*x*‐dir: 0.325	0.012	0.180	15.69	0.223	4.32
		*y*‐dir: 0.193	0.013	0.104	51.50	0.141	34.36
		*z*‐dir: 0.169	0.002	0.109	49.41	0.114	46.55

### Geometric tortuosity

**Figure 5:**
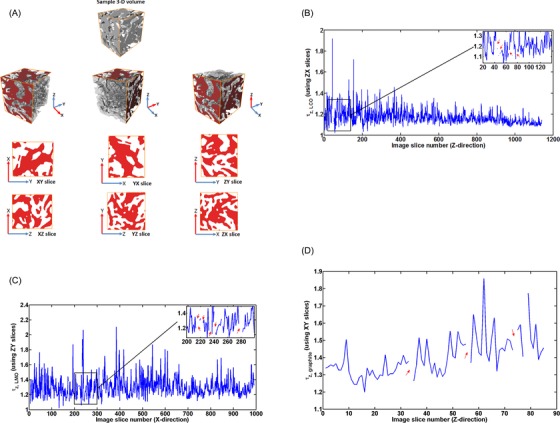
(A) Schematic illustration of how a 3‐D structure can be viewed along each axis direction for 2‐D tortuosity calculations. Slicewise tortuosity profile in the pore phase across (B) ZX image sections in the LCO electrode, (C) ZY image sections in the LMO electrode and (D) XY image sections in the graphite electrode. Regions of tortuosity discontinuity highlighted by the red arrows in the inset plots signify no pore percolation across some image section(s) within the region of the sample.

Although the arc‐chord ratio for calculating geometric tortuosity is not a relationship developed from stereological theory, it has been used on 2‐D image sections for estimating the tortuosity of inherently 3‐D structures (Onkaew *et al*., 2011). Moreover, as illustrated in Figure 5(A) using a small ROI from the reconstructed LCO electrode pore network, 2‐D image based estimations of the tortuosity of a 3‐D pore network along a given orthogonal direction means that the 2‐D planar images for this could be viewed or selected from two possible directions. For example, if we are estimating tortuosity along the *z* direction in a tomographically generated 3‐D image using the arc‐chord ratio approach, the tortuosity estimates can be calculated using 2‐D image sections generated in the *ZX* or *ZY* planes, and in the *x* and *y* directions if the 3‐D image is re‐sliced to generate image sections in the *XZ* or *XY* planes and *YX* or *YZ* planes, respectively. This results in two tortuosity profiles per axial direction, making a total of six possible tortuosity profiles. Although the pore phase in the examined electrodes showed good connectivity (>99 %) and percolation in three dimensions, we observe that the pore phase across some individual 2‐D sections generated was not connected, leading to discontinuities in the slicewise tortuosity profiles as the tortuosity values across such sections is taken to be infinite.

**Figure 6:**
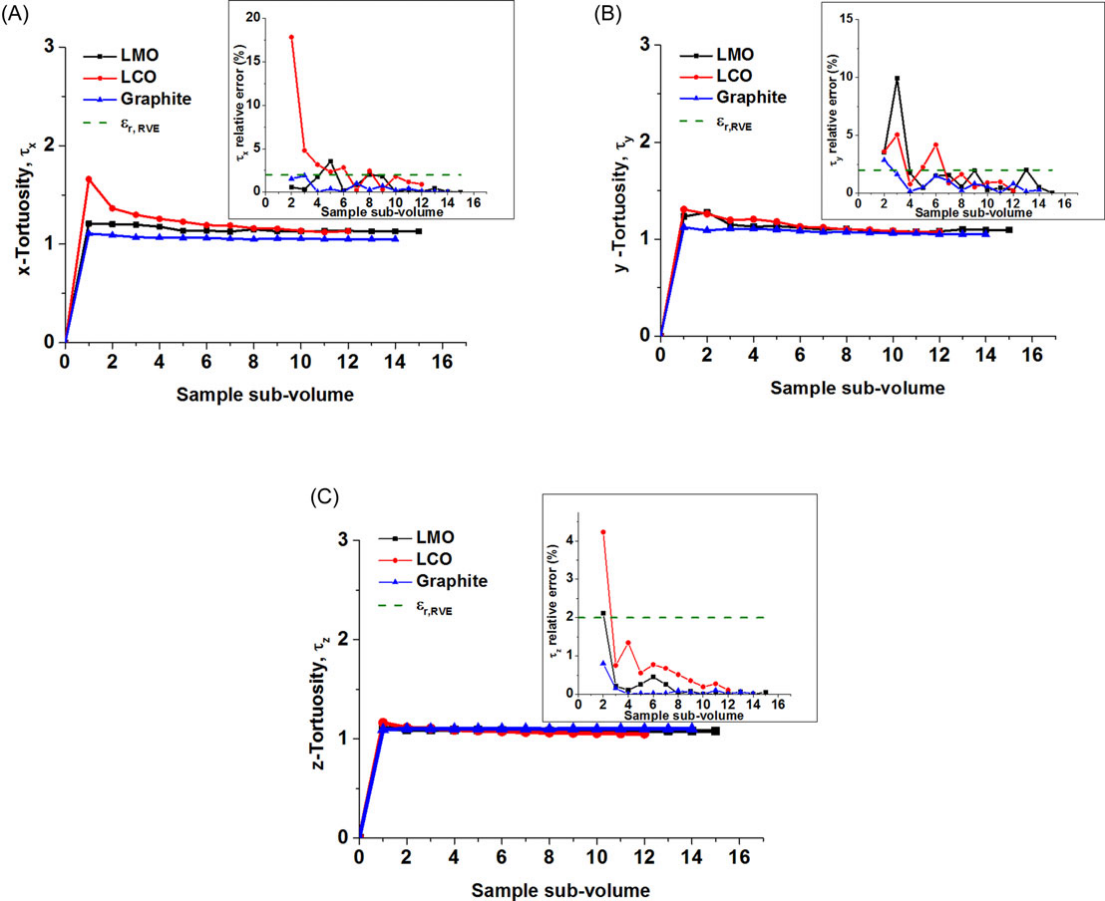
Evolution of 3‐D pore‐phase tortuosity along each orthogonal direction (main) and the relative errors (inset) versus subvolume size for each electrode sample.

Figure 5(B‐D) show the discontinuities in the slicewise tortuosity profile for each of the examined electrodes, which highlights the absence of a completely percolating pore phase across some 2‐D sections. These discontinuities were present in all the 2‐D tortuosity slicewise profiles created along each possible plane. Figure 6 shows the evolution of 3‐D pore‐phase tortuosity with increasing sample subvolume along the x, y and z directions using representative volume element analysis. The discontinuities in Figure 5 show that the tortuosity within inherently 3‐D networks is nearly impossible to measure from 2‐D cross‐sections or planar slices, as it relies on exactly how and where the phase networks branch out and interconnect in three dimensions (Wilson *et al*., 2006; Wilson *et al*., 2011); the 2‐D methodology does not consider the 3‐D phase network interconnectivity, and hence such an approach for estimation of 3‐D tortuosity is bound to be associated with ambiguity. Table 5 compares the stereological mean *z*‐direction tortuosity (obtained using *ZX* image sections) with the directional tortuosities obtained directly from the 3‐D volumes. Here, the stereological mean *z*‐direction tortuosities in each electrode sample appear to be largely overestimated because they are much higher than the *z*‐direction tortuosities obtained from 3‐D analysis. This overestimation is most likely associated with the fact that the shortest pathway through such heterogeneous pore networks from a 2‐D cross‐section is actually a much shorter route when the entire 3‐D pore network is considered.

**Table 5:** Comparison of geometric tortuosity obtained by stereological predictions and 3‐D analysis

Geometric tortuosity, τ							
Sample	3‐D	Mean 2‐D (*z*‐dir, *ZX* slices)	SD 2‐D (*z*‐dir, *ZX* slices)	Min 2‐D (*z*‐dir, *ZX* slices)	% UE	Max 2‐D (*z*‐dir, *ZX* slices)	% OE
LCO	$\tau_{x}$: 1.078 $\tau_{y}$: 1.060 $\tau_{z}$: 1.011	1.170	0.069	1.023	1.18	1.919	89.82
Graphite	$\tau_{x}$: 1.051 $\tau_{y}$: 1.054 $\tau_{z}$: 1.013	1.485	0.147	1.167	2.736	21.42	84.26
LMO	$\tau_{x}$: 1.069 $\tau_{y}$: 1.053 $\tau_{z}$: 1.010	1.225	0.105	1.077	6.61	1.936	91.6

### Pore radius

**Figure 7:**
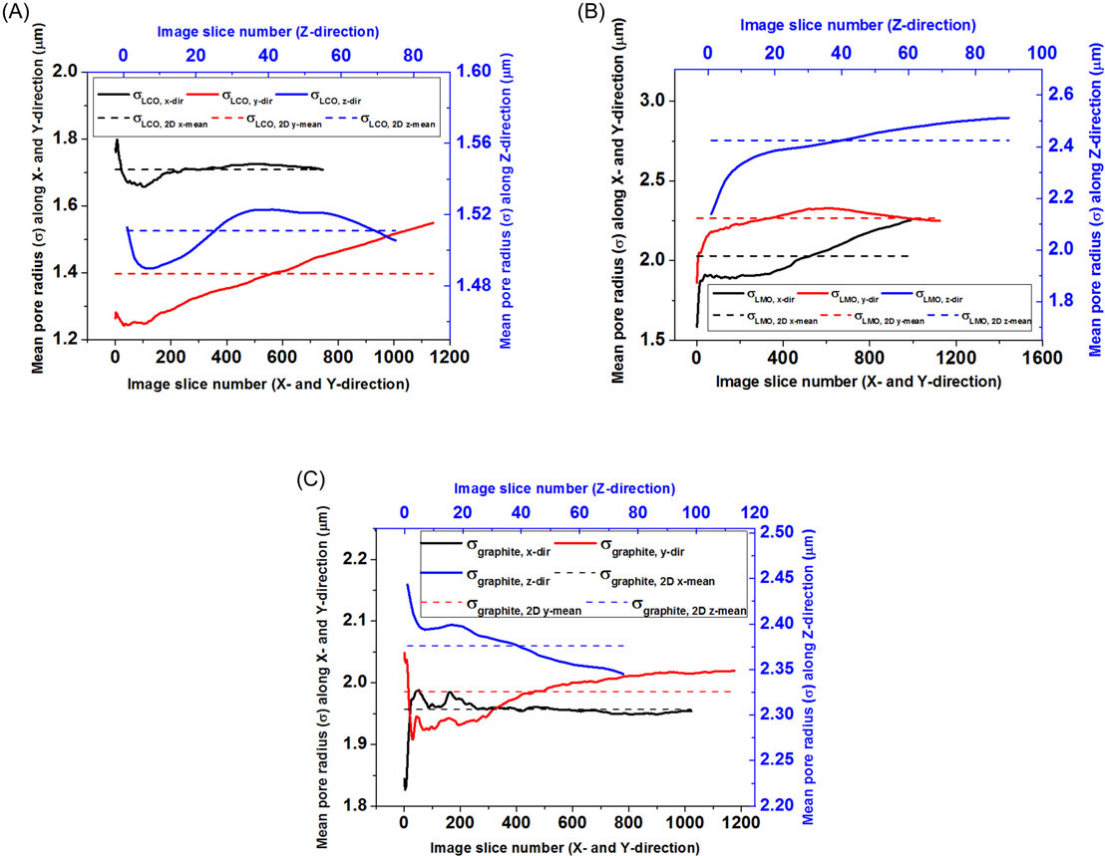
Mean pore radius profiles for (A) LCO, (B) LMO and (C) graphite electrode samples showing variation in slicewise pore radii along the *x*, *y* and *z* directions.

With the stereological approach, slicewise profiles for mean pore radius were generated for each electrode sample using 2‐D sections generated along each axis. In all three samples, significant variations in pore radius are seen in each slicewise profile along each axis. Moreover, in all three electrode samples, the mean pore radius values obtained along each axis direction appear to be in close range (Fig. 7 and Table 6);

**Table 6:** Comparison of pore radius obtained from stereological prediction and different 3‐D analysis methods

Pore radius, σ							
Sample	3‐D (μm)	Mean 2‐D (μm)	SD 2‐D (μm)	Min 2‐D % OE	% UE	Max 2‐D (μm)	% OE
LCO	DOHT: 1.049	*x*‐dir: 1.708	0.021	1.656	27.26	1.797	21.07
	MA‐FM: 2.251	*y*‐dir: 1.397	0.092	1.243	45.43	1.549	31.99
	CPSD: 2.277	*z*‐dir: 1.511	0.011	1.490	34.56	1.523	33.12
	SMO: 2.394						
Graphite	DOHT: 1.650	*x*‐dir: 1.957	0.015	1.827	48.25	1.988	43.70
	MA‐FM: 3.531	*y*‐dir: 1.986	0.032	1.908	45.96	2.050	41.98
	CPSD: 3.031	*z*‐dir: 2.376	0.021	2.345	33.58	2.443	30.80
	SMO: 3.155						
LMO	DOHT: 2.723	*x*‐dir: 2.030	0.218	1.586	53.48	2.254	33.86
	MA‐FM: 3.680	*y*‐dir: 2.265	0.060	1.863	45.34	2.330	31.64
	CPSD: 3.408	*z*‐dir: 2.425	0.073	2.140	37.20	2.512	26.29
	SMO: 3.443						

**Figure 8:**
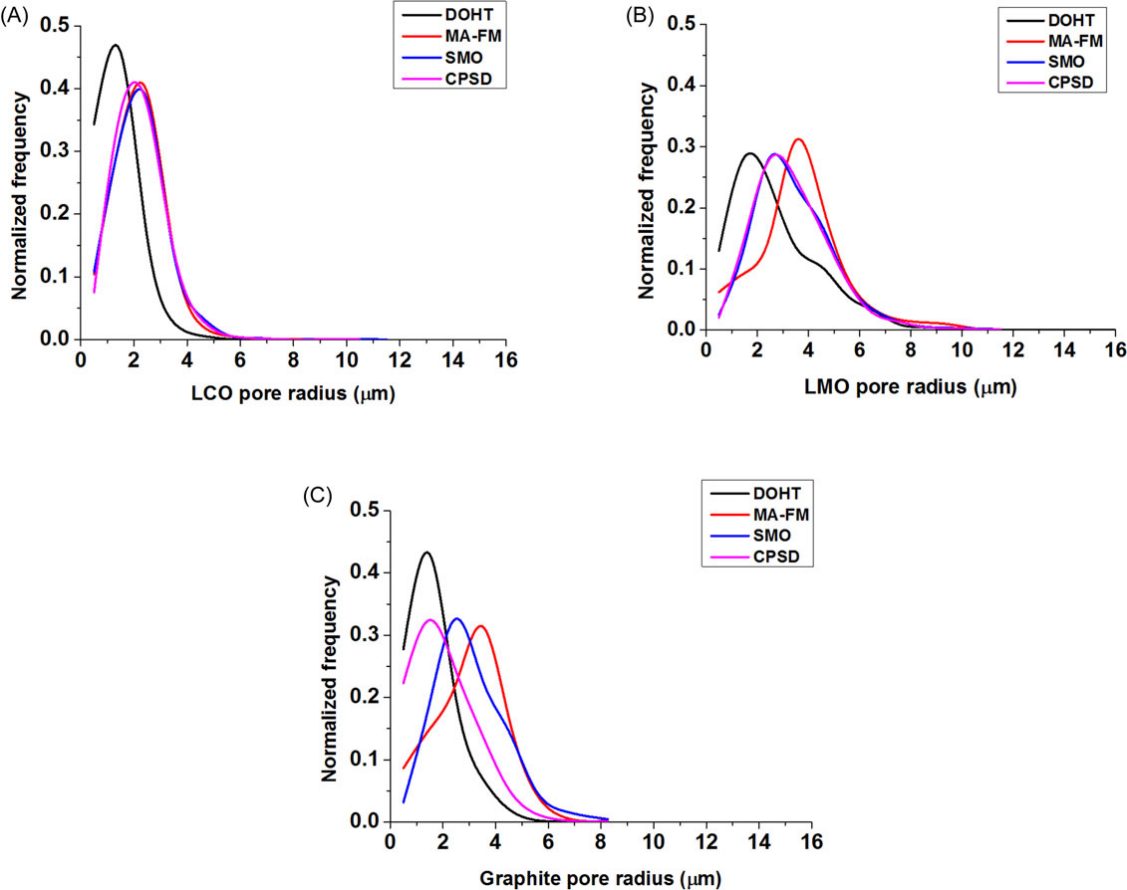
Pore radius distribution of the battery electrode samples (A) LCO, (B) LMO and (C) graphite.

With regards to the 3‐D reconstructed volumes, four different approaches for extracting the mean pore radius parameter were examined, as outlined previously. Figure 8 shows a comparison of the pore radius distributions generated from these methods. Of all three electrode samples, the LMO electrode appears to have the broadest pore size distribution and the highest mean pore radius value. It is also observed that the DOHT method gives a high frequency of smaller pores ‐ this is associated with the tendency of the thinning algorithm to generate false branches and spurious nodes in the resulting skeleton, which are often induced by surface irregularities and image noise. However, these can be eliminated by pruning the false branches within the skeleton but this is a nontrivial process for such complex microstructures and could end up significantly altering the original topological skeleton.

The MA‐FM method, however, appears to have a relatively higher frequency of larger pores. This is as a result of the characteristic behaviour of the fast marching algorithm employed to perform selective pore radius sampling on larger pore pathways rather than on every pore pathway due to the scaling of the speed map with the interfacial distance map (Jørgensen *et al*., 2011). The pore radius distribution generated with the MA‐FM, SMO and CPSD methods are almost identical for the LCO sample. In both the LMO and LCO samples, the pore radius distribution curves from the CPSD and SMO methods are similar. However, this trend is absent in the graphite electrode sample, with the SMO method displaying a broad pore radius distribution.

It is noteworthy to mention that pore‐scale quantification of tomography data has been extensively validated against conventional porosimetry techniques (e.g. Safinia *et al*., 2006; Jones *et al*., 2007; Izzo *et al*., 2008); moreover, the CPSD algorithm was developed to mimic the physical process of mercury porosimetry to extract values from tomography data that can be directly compared with porosimetry measurements.

Although these 3‐D pore size extraction methods are commonly used in pore‐scale characterization of porous materials, each of them has its advantages and drawbacks (e.g. DOHT method is easily affected by image noise and could create false skeleton branches and nodes, whereas the MA method selectively samples pore pathways) and the differences in calculated pore radius values suggests that there stands the risk of confusion about the geometric definition of pore size and size distribution. However, the CPSD method would be expected to have a high degree of accuracy as it includes all possible pathways including narrow bottlenecks and dead‐ended pores, and is not limited by possible errors from topological skeleton creation.

Therefore, it is recommended that one proceed with caution when selecting amongst these pore size calculation methods for quantifying complex porous microstructures, and consequently any comparison of 3‐D pore size distribution should fully account for inherent difference that may be present in the calculation methodology. Moreover, even as all pore size calculations should be independent of imaging resolution (as the digital image resolution dependencies decrease once the voxel size falls below that of the structures to be resolved), it is useful to image the porous material of interest at a sufficiently high resolution to reduce the effect of possible artefacts from image processing. Applying a multiscale 3‐D imaging approach (Shearing *et al*., 2012) will provide a good validation for microstructural quantification. Furthermore, when the stereological mean pore radii values were compared with the mean pore radius values obtained using the CPSD method, large underestimations (and overestimations) of over 20 % were observed.

## Conclusion

In this study, both stereological prediction and 3‐D analysis techniques for quantitative assessment of key geometric parameters for characterizing lithium‐ion battery electrode microstructures were examined and compared. Quantitative analysis was carried out on image data obtained from imaging battery electrode samples using synchrotron‐based X‐ray tomographic microscopy. For each electrode sample investigated stereological analysis was performed on 2‐D planar image slices generated from tomographic imaging, whereas direct 3‐D analysis was performed on rendered image volumes; representative volume element analysis was performed to ascertain the selected 3‐D volumes examined were representative of the bulk electrode.

The results showed that stereological estimation of inherently 3‐D microstructural parameters using a single 2‐D image section is bound to be associated with ambiguity and may lead to significant parameter under‐ or overestimation. Significant variation in measured parameters observed using 2‐D planar image sections highlights the presence of localised microstructural heterogeneities within the electrode materials. Pore volume fraction measurements using stereological prediction showed smaller parameter variations using planar slices normal to the *z* direction; in this case, stereology could be used to obtain an initial approximation for pore volume fraction but in order to obtain complete information on a statistically, nonhomogenous microstructure, direct 3‐D measurement cannot be replaced by stereological relationships. Discontinuities in the planar tortuosity profiles show that the tortuosity within inherently 3‐D networks cannot be geometrically measured from 2‐D cross‐sections or planar slices, as tortuosity relies on exactly how and where the phase networks branch out and interconnect in three dimensions. This also demonstrates that 3‐D measurement of nonconvex, irregular and interconnected pore networks are more suited for accurately quantifying spatial parameters, like tortuosity and phase connectivity. Although there is bias introduced when applying stereological relationships to measure inhomogeneous 3‐D microstructures, direct 3‐D measurements have the demerit of being more computationally intensive.

We also compare four different methods to extract pore size distributions in 3‐D. The CPSD and SMO methods give similar pore‐size distributions curves as they consider the entire network including dead‐ended and isolated pores, as opposed to the MA‐FM method which tends to sample major pore pathways. It is recommended that comparison of 3‐D pore size distributions should fully account for inherent difference that may be present in each calculation methodology.
